# Supplementary material for: Robust multi‐coil MRI reconstruction via self‐supervised denoising
Source: Magn Reson Med. 2025 Jun 2;94(5):1859–77. doi: 10.1002/mrm.30591 (PMC12354090; doi:10.1002/mrm.30591)
Supplement: Supplementary file 1 — Data S1. Supporting Information. [file MRM-94-1859-s001.docx]

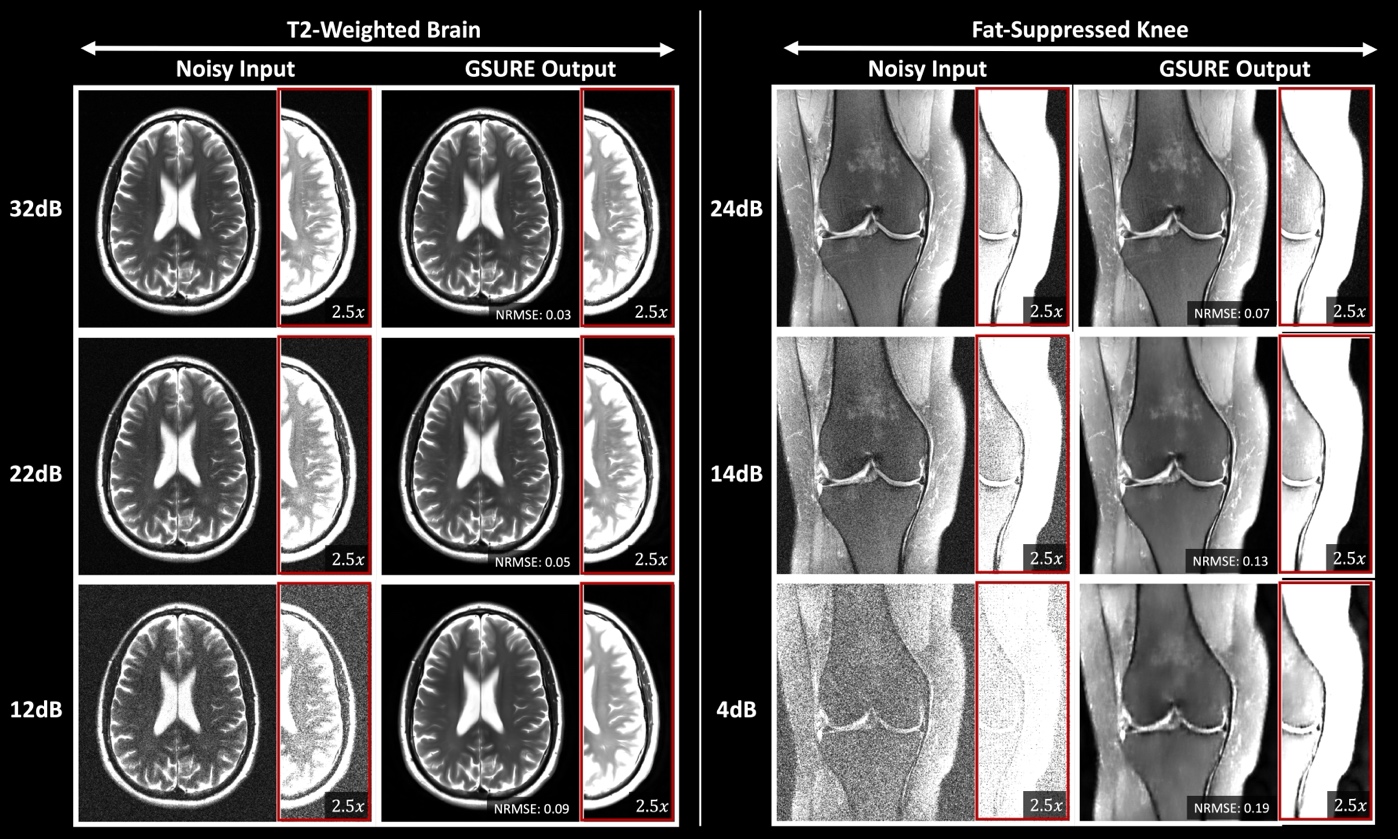


Figure S1: Validation examples from the GSURE denoising experiment across T2-Weighted Brain and Fat-Suppressed Knee data at three SNR levels. Across each column under the appropriate anatomy, we show noisy input vs the output of the GSURE network. Across each row under the appropriate anatomy, we show the same validation example at different SNR levels, where row 1 always shows data at the original native SNR (without additive noise). Overall, the experiment showcases the utility of GSURE denoising in improving the quality of training data, especially in low SNR cases.


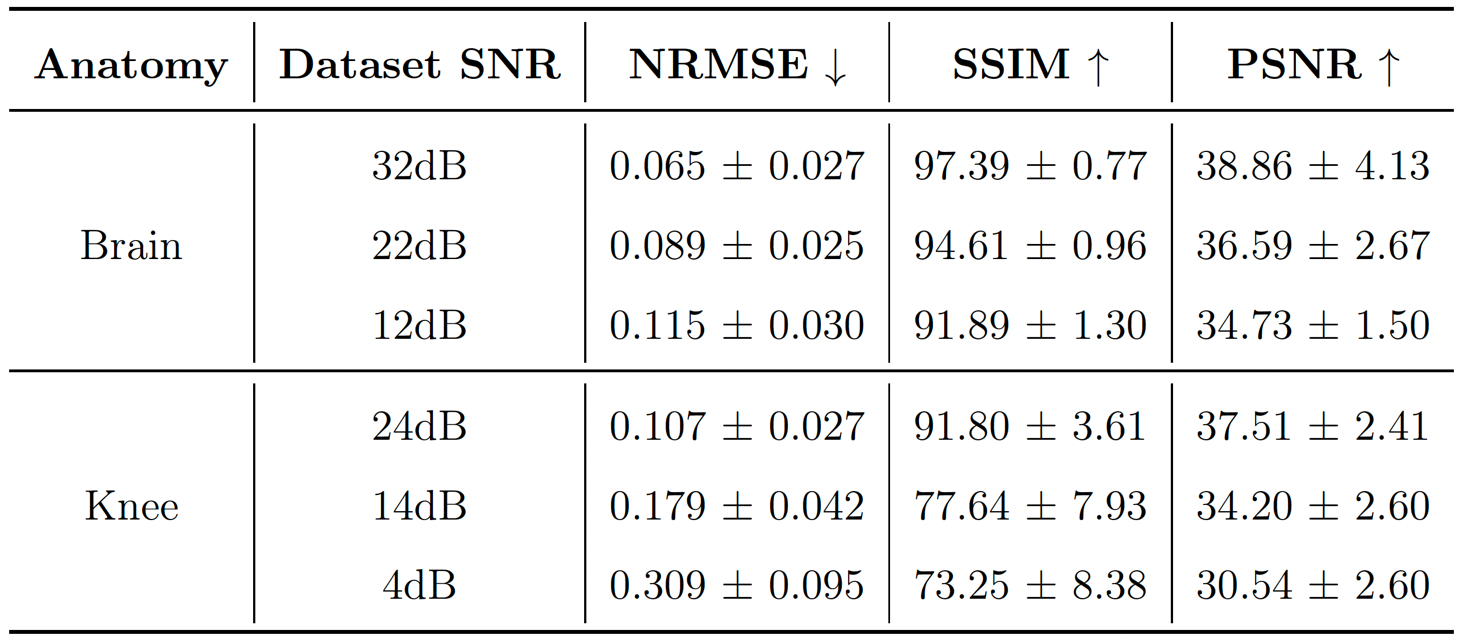


Table S1: Validation performance of GSURE denoising averaged across 100 examples, for three SNR levels and two anatomies: a) T2-Weighted Brain and b) Fat-Suppressed Knee.


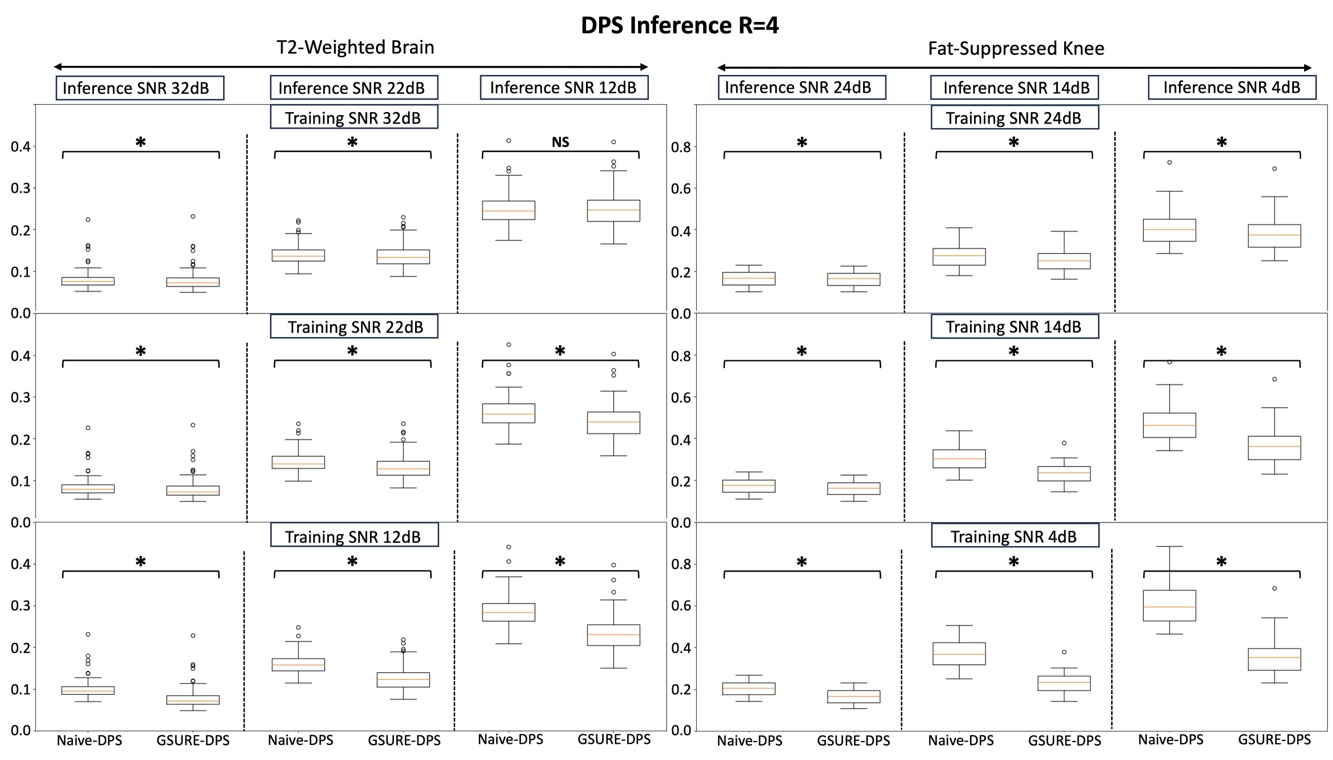


Figure S2: Box plot of 100 validation reconstruction examples (NRMSE) comparing Naive-DPS and GSURE-DPS at acceleration factor R=4, across: a) T2-Weighted Brain and Fat-Suppressed anatomies, b) training SNR levels, c) inference SNR levels. (*statistically significant difference in reconstruction performance, NS: not significant).


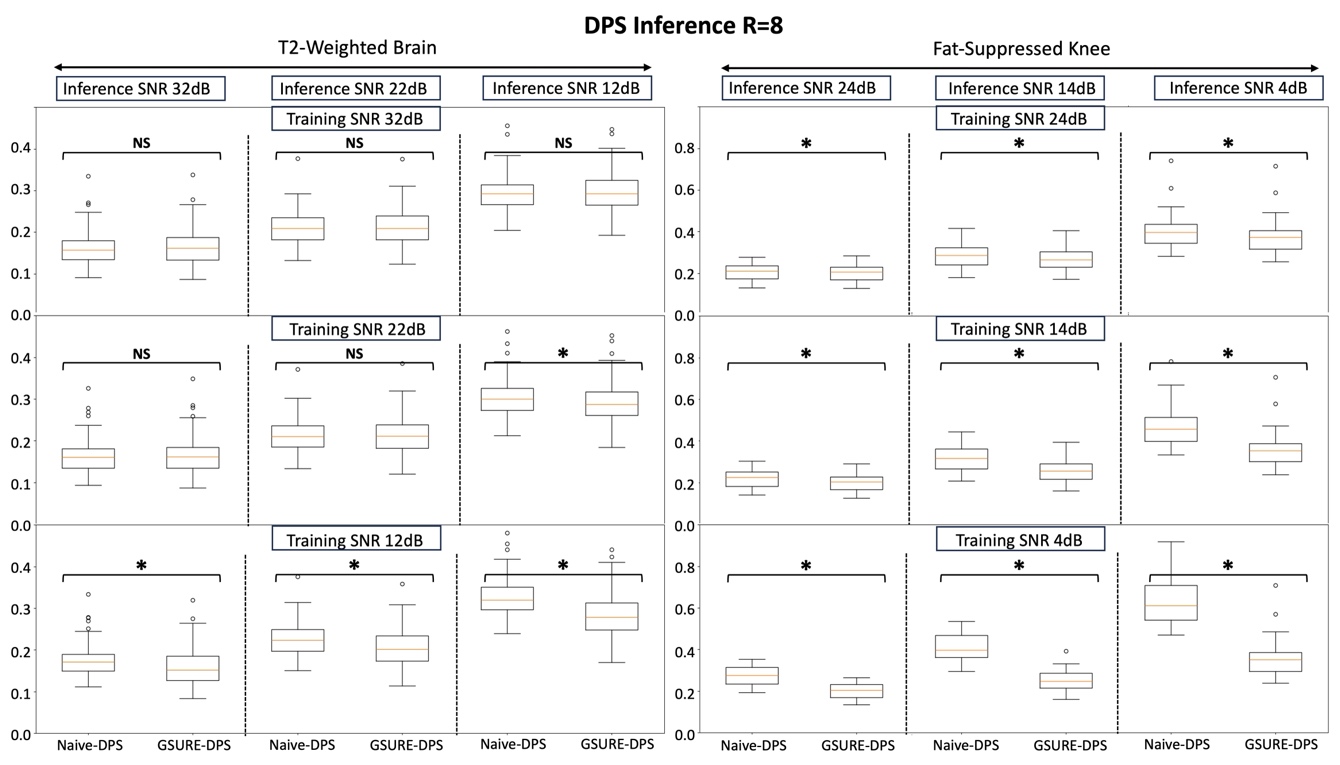


Figure S3: Box plot of 100 validation reconstruction examples (NRMSE) comparing Naive-DPS and GSURE-DPS at acceleration factor R=8, across: a) T2-Weighted Brain and Fat-Suppressed anatomies, b) training SNR levels, c) inference SNR levels. (*statistically significant difference in reconstruction performance, NS: not significant).


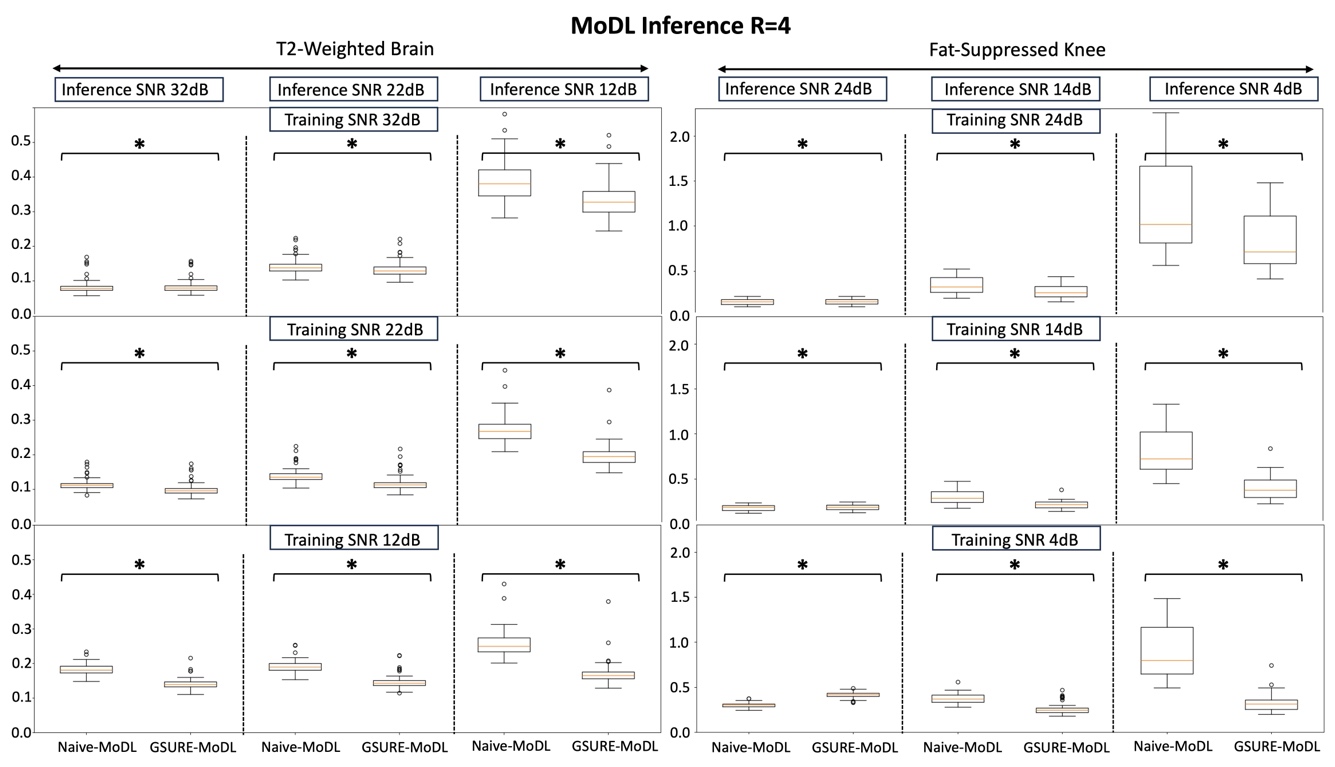


Figure S4: Box plot of 100 validation reconstruction examples (NRMSE) comparing Naive-MoDL and GSURE-MoDL at acceleration factor R=4, across: a) T2-Weighted Brain and Fat-Suppressed anatomies, b) training SNR levels, c) inference SNR levels. (*statistically significant difference in reconstruction performance, NS: not significant).


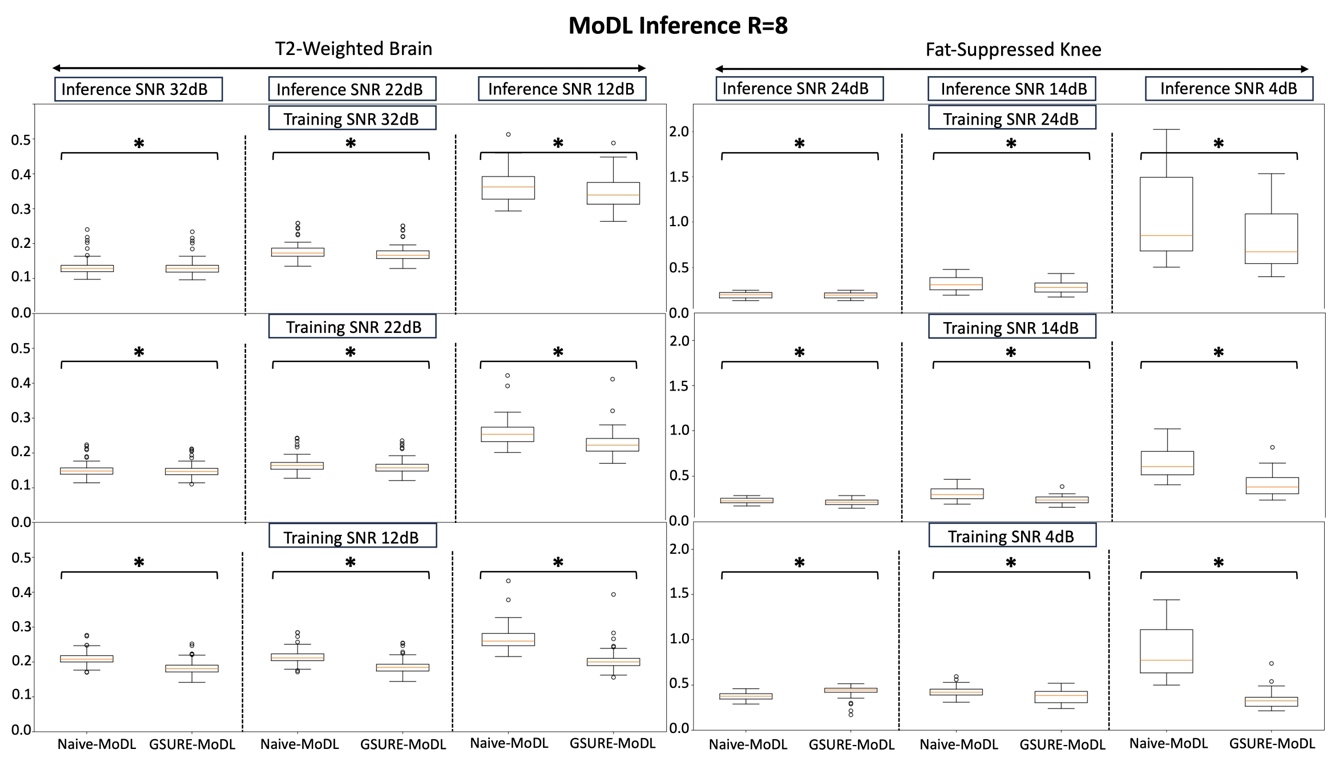


Figure S5: Box plot of 100 validation reconstruction examples (NRMSE) comparing Naive-MoDL and GSURE-MoDL at acceleration factor R=8, across: a) T2-Weighted Brain and Fat-Suppressed anatomies, b) training SNR levels, c) inference SNR levels. (*statistically significant difference in reconstruction performance, NS: not significant).


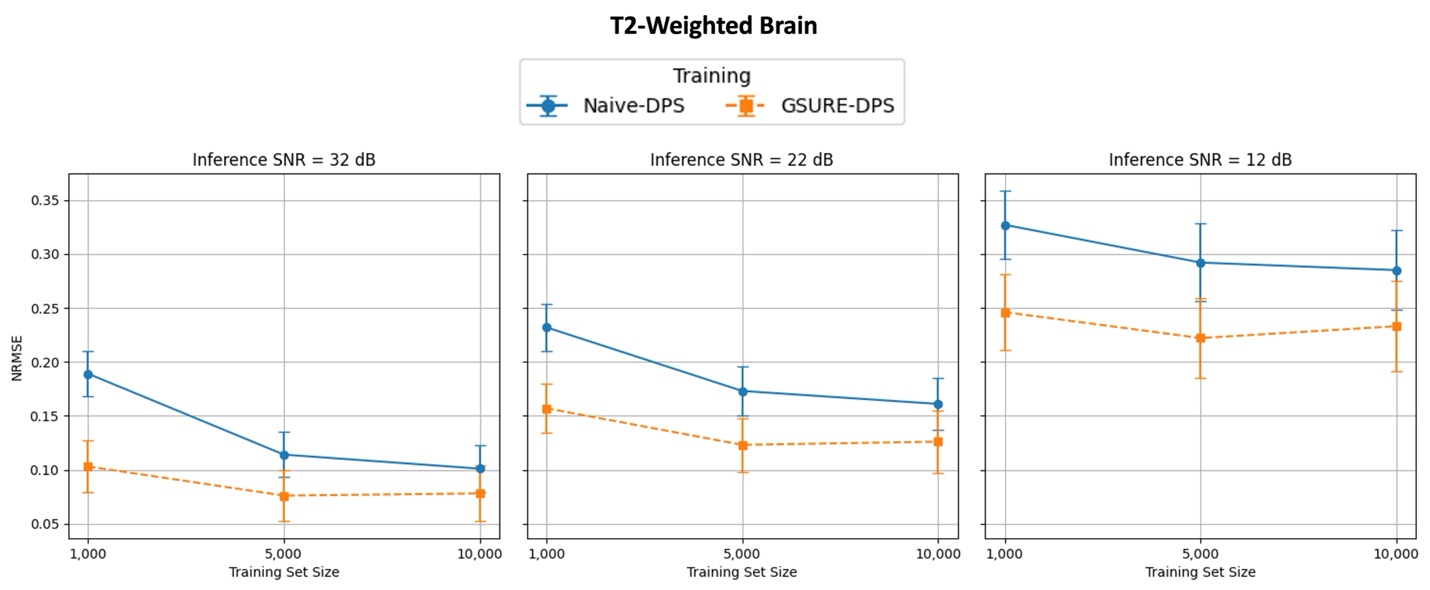


Figure S6: Reconstruction performance comparison between Naive-DPS and GSURE-DPS trained on T2-Weighted Brain dataset at 12dB SNR and three different training set sizes. Each plot compares Naive-DPS and GSURE-DPS, where each point on the line represents: a) on y-axis the average NRMSE across 100 validation examples (averaged across 5 random seeds), and b) on x-axis: the training dataset size for the experiment. We can observe that GSURE-DPS reconstructions consistently require less training samples to achieve a target NRMSE, showcasing that GSURE-DPS can improve reconstruction quality and enable faster training.


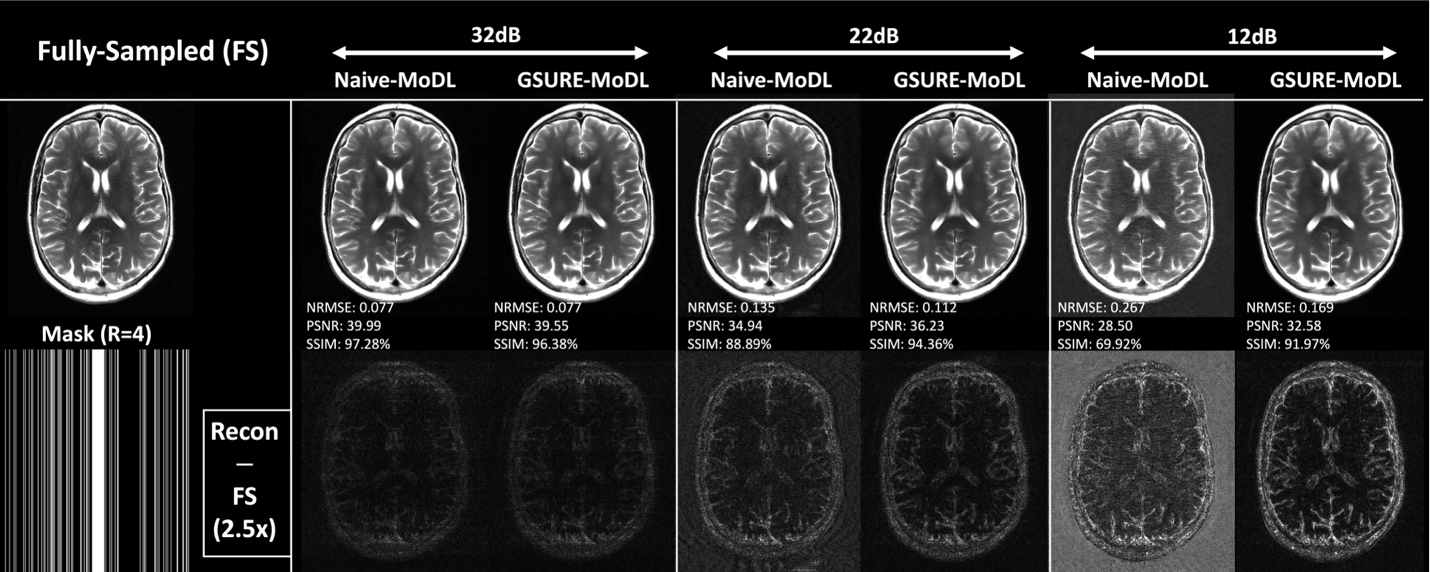


Figure S7: T2-Weighted Brain reconstructions with MoDL, utilizing MoDL models trained on two datasets: a) Noisy (Naive-MoDL) and b) GSURE denoised (GSURE-MoDL). Across columns, we show reconstructions across three training/inference SNR levels. In the first row, we show the reconstruction example with quantitative comparison metrics. In the second row, we show the difference between the reconstruction and fully sampled image at 2.5x brightness. We can observe that GSURE-MoDL outperforms Naive-DPS notably at lower SNR levels.


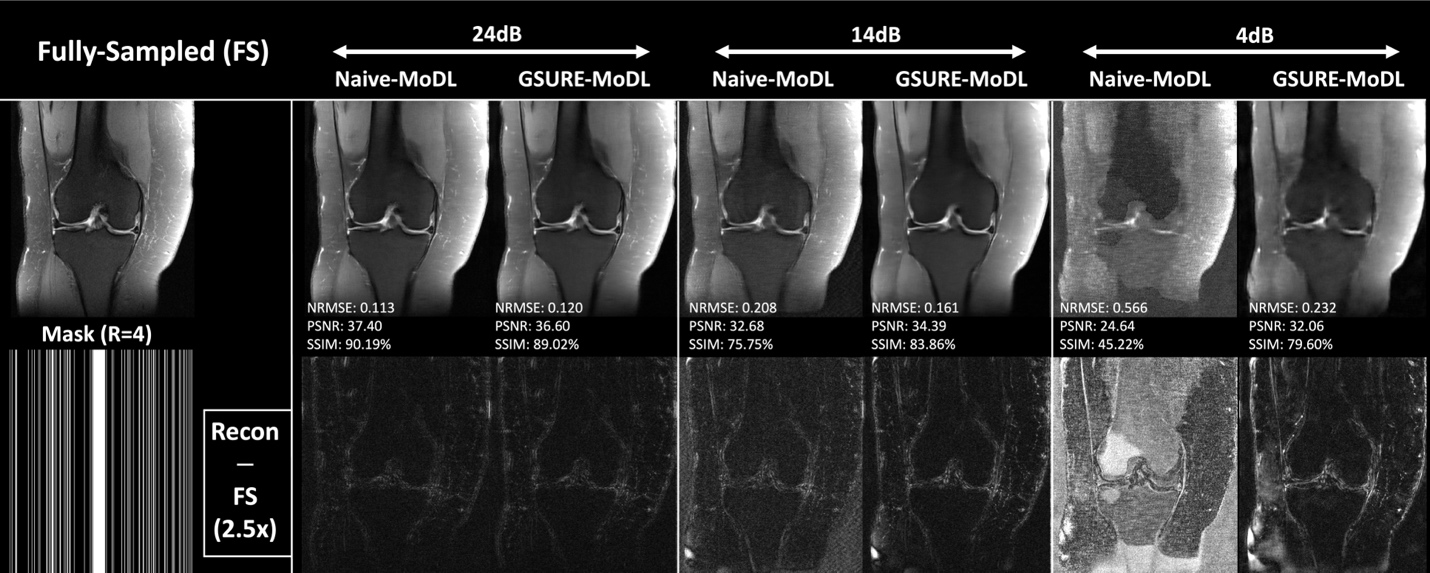


Figure S8: Fat-Suppressed Knee reconstructions with MoDL, utilizing MoDL models trained on two datasets: a) Noisy (Naive-MoDL) and b) GSURE denoised (GSURE-MoDL). Across columns, we show reconstructions across three training/inference SNR levels. In the first row, we show the reconstruction example with quantitative comparison metrics. In the second row, we show the difference of the reconstruction and fully sampled image at 2.5x brightness. We can observe that GSURE-MoDL outperforms Naive-DPS notably at lower SNR levels.


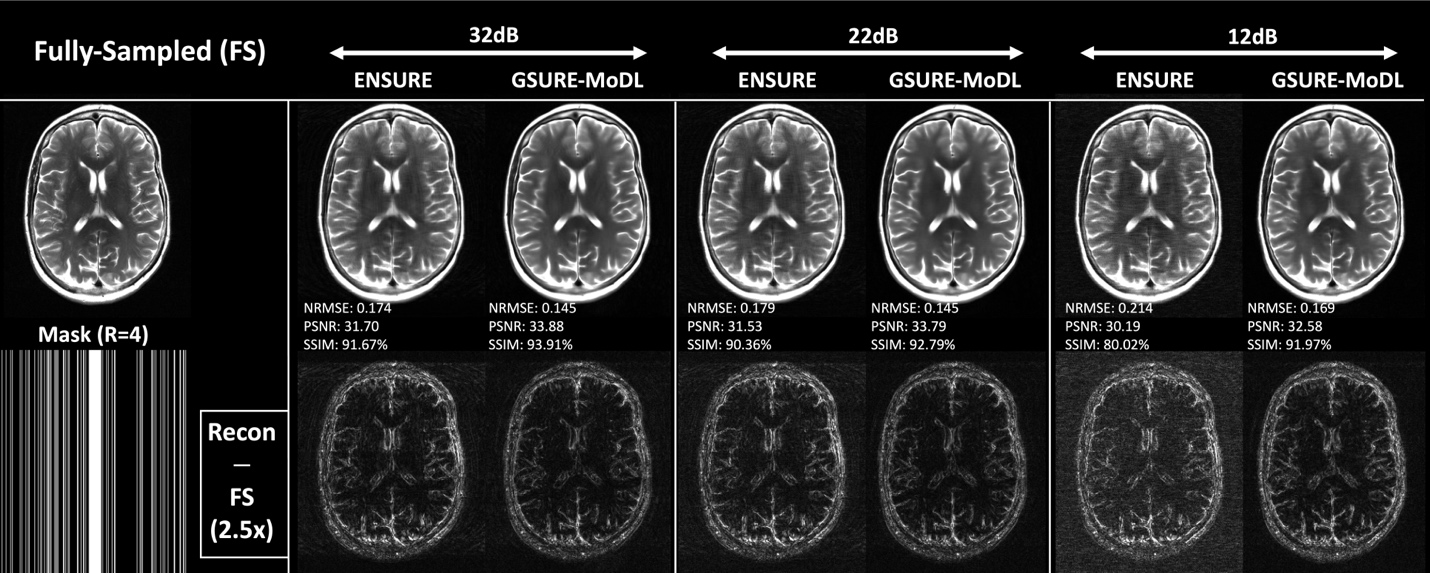


Figure S9: T2-Weighted Brain reconstructions with ENSURE and GSURE-MoDL models trained on 12dB SNR data. Across columns, we show reconstructions across three inference SNR levels. In the first row, we show the reconstruction example with quantitative comparison metrics. In the second row, we show the difference between the reconstruction and fully sampled image at 2.5x brightness. We can observe that GSURE-MoDL outperforms ENSURE, notably at lower SNR levels.


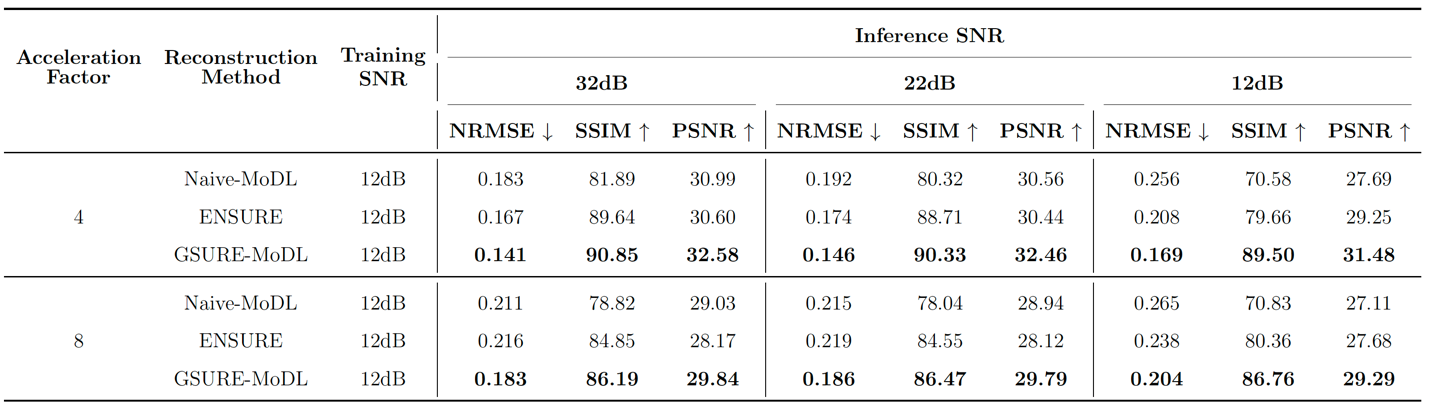


Table S2: T2-Weighted Brain reconstruction metrics in comparison to ENSURE. Across each inference SNR, acceleration factor, and reconstruction method, we **highlight** the training strategy with the best reconstruction performance (lowest error).
